# SroA links SigS-dependent stress signaling to metabolic remodeling in *Staphylococcus aureus*

**DOI:** 10.64898/2026.05.15.725384

**Published:** 2026-05-15

**Authors:** Shahad Alqahtani, Sagar Pasham, Jamilah Alsulami, Amer Al Ali, Joseph I Aubee, Brooke R. Tomlinson, Sarah J. Kennedy, Emily Felton, Lindsey N. Shaw, Karl M Thompson

**Affiliations:** 1 Department of Microbiology, College of Medicine, Howard University, 520 W Street, NW, Suite 3010, Washington, DC 20059, USA; 2 Department of Biological Sciences, Faculty of Science and Humanities, Shaqra University, P. O. Box 1040, Ad-Dawadimi 11911, Saudi Arabia; 3 Current address: Saudi Food and Drug Authority, Saudi Arabia; 4 Current address: Department of Medical Laboratory Science, College of Applied Medical Sciences, University of Bisha, Saudi Arabia; 5 Department of Molecular Biosciences, University of South Florida, 4202 East Fowler Ave, ISA2015, Tampa, FL 33620-5150, USA; 6 Center for Antimicrobial Resistance, University of South Florida, 4202 East Fowler Ave, ISA2015, Tampa, FL 33620-5150, USA

**Keywords:** SroA, SigS, DUFs, *narG*, *nar* operon, nitrate metabolism, *S. aureus*

## Abstract

*Staphylococcus aureus* encounters diverse environmental conditions during colonization and infection, including fluctuations in nutrient availability, oxidative stress, and oxygen limitation. Adaptation to these environments requires regulatory systems that coordinate stress responses with metabolic remodeling. The extracytoplasmic function sigma factor SigS contributes to stress adaptation and virulence in *S. aureus* and directly activates expression of the *sroAB* operon, which encodes the small proteins SroA and SroB. While previous work demonstrated that SroA participates in feedback regulation of *sigS* expression, the broader physiological role of SroA has remained unclear. To define the regulatory functions of SroA, we performed RNA sequencing following inducible overexpression of *sroA* in *S. aureus*. Transcriptome analysis revealed extensive remodeling of gene expression, with approximately 200 transcripts significantly altered. Transcriptome analysis revealed coordinated repression of metabolic pathways (including nitrate respiration and nucleotide biosynthesis) alongside activation of stress-response and nutrient acquisition genes. Northern blot and quantitative RT-PCR analysis confirmed repression of *narG* and *narJ* transcripts following SroA overexpression. Consistent with these transcriptional changes, nitrate reduction assays demonstrated that SroA overexpression reduces nitrate respiration activity. In addition to repression of nitrate respiration genes, SroA overexpression broadly suppressed genes involved in de novo purine and pyrimidine biosynthesis. In contrast, transcripts associated with stress responses and nutrient acquisition, including the SOS-associated gene *sosA* and the phosphate transport gene *pstS*, were upregulated. Together, these findings identify SroA as a regulator that links stress-responsive signaling to metabolic remodeling in *S. aureus*, particularly through modulation of nitrate respiration pathways.

## Importance

*Staphylococcus aureus* must rapidly adapt its metabolism to survive the diverse environments encountered during colonization and infection, including conditions where oxygen availability is limited. In this study, we identify a previously uncharacterized role for the small protein SroA in regulating metabolic adaptation in *S. aureus*. Transcriptome analysis revealed that SroA strongly represses genes involved in nitrate respiration, a pathway that enables bacteria to maintain energy production when oxygen is scarce. Consistent with these transcriptional changes, SroA overexpression reduced nitrate respiration activity. These findings reveal a regulatory link between stress-responsive signaling pathways and respiratory metabolism, expanding our understanding of how *S. aureus* adapts to oxygen-limited environments encountered during infection.

## Introduction

*Staphylococcus aureus* is a Gram-positive bacterium within the phylum Firmicutes that functions both as a commensal colonizer and an opportunistic human pathogen. This organism commonly inhabits the anterior nares and skin of healthy individuals but can cause a wide range of infections when host barriers are breached (1). These infections range from superficial skin and soft tissue infections to invasive diseases affecting deep tissues, the respiratory tract, gastrointestinal tract, and bloodstream. The emergence of methicillin-resistant *S. aureus* (MRSA) strains has further increased the clinical burden associated with this pathogen.

During colonization and infection, *S. aureus* encounters diverse environmental conditions within host tissues, including fluctuations in nutrient availability, oxidative stress, and oxygen limitation. Adaptation to these environments requires rapid remodeling of metabolic and stress-response programs that allow the bacterium to maintain growth and survival under changing conditions. Oxygen limitation within host tissues can necessitate shifts in respiratory metabolism, including the use of alternative electron acceptors such as nitrate to support redox balance and energy production. Regulatory systems that coordinate gene expression in response to environmental cues are therefore essential for bacterial adaptation and virulence (2).

These adaptive responses are coordinated by complex regulatory networks that control both virulence and metabolic pathways in *S. aureus* (2). These networks include numerous transcriptional regulators and two-component signaling systems that modulate gene expression in response to environmental cues encountered during colonization and infection (2). Among these regulatory mechanisms, alternative sigma factors play important roles in redirecting RNA polymerase to specific promoters during environmental and host-associated stresses or nutrient limitation. There are three alternative sigma factors in *S. aureus*: SigB, SigH, and SigS. SigB functions in stationary phase and general stress adaptation and influences virulence factor, toxin, and drug resistance gene expression (3–6). SigH contributes to competence-associated gene regulation (7–10). SigS, an extracytoplasmic function (ECF) sigma factor, has emerged as an important regulator of stress adaptation and virulence (7).

Expression of SigS is induced in response to genotoxic stress, including DNA damage caused by acidic pH and exposure to the DNA-damaging agent methyl methanesulfonate (MMS) (7). Consistent with this role, SigS contributes to bacterial survival under conditions that challenge cellular integrity, including genotoxic stress, cell wall stress, and host immune defenses (7, 8). Regulation of *sigS* occurs through a complex transcriptional architecture involving multiple promoters, transcription factors, and an antisense transcript (9, 10). Previous studies identified the *sroAB* operon as a direct transcriptional target of SigS (11). The *sroAB* operon encodes two small proteins, SroA and SroB, that exert divergent regulatory effects on *sigS* expression, suggesting that these proteins participate in feedback regulation of SigS-dependent signaling (11). However, the broader physiological role of SroA and its contribution to stress-responsive regulatory networks remain largely unknown. While SigS-dependent regulation has been linked to stress adaptation, how downstream effectors coordinate metabolic remodeling in response to stress remains poorly defined.

To define the physiological role of SroA in *S. aureus*, we performed transcriptomic analysis following inducible expression of *sroA*. RNA sequencing revealed that SroA overexpression drives extensive remodeling of gene expression across multiple metabolic and stress-responsive pathways. Among the most strongly repressed transcripts were genes involved in nitrate respiration, including the nitrate reductase operon. Consistent with these observations, SroA overexpression resulted in repression of the nitrate reductase operon and corresponding reductions in nitrate respiration activity. In addition to these metabolic changes, transcripts associated with nucleotide biosynthesis were broadly repressed, whereas stress-responsive genes including the SOS-associated cell division inhibitor *sosA* and components of the phosphate transport system were upregulated. Together, these findings identify SroA as a previously unrecognized regulator that links stress-responsive signaling to metabolic remodeling and respiratory adaptation in *S. aureus*.

## Materials and Methods

### Media and Growth Conditions.

All *S. aureus* cells were grown in 5 mL of Tryptic Soy Broth (TSB) (KD Medical, Columbia, MD, USA) overnight in a Cel-Gro Tissue Culture Rotator (Fisher Scientific, Waltham, MA, USA) at 37°C in a SMI12 microbiological incubator (Sheldon Manufacturing, Cornelius, OR). For gene expression and molecular biological analysis under aerobic conditions, overnight cultures were diluted 1:500 to 1:1000 in 30 to 50 mL of TSB in a 125 mL or 250 mL beveled Erlenmeyer Flask and incubated in a WS27 shaking water bath (Sheldon Manufacturing, Cornelius, OR, USA). TSB was supplemented with antibiotics, xylose, or sodium nitrate as needed for experiments described in this study. For antibiotics, TSB was supplemented with chloramphenicol to a final concentration of 10 μg / mL to select for plasmids used in this study.

#### Induction of SroA Expression.

To induce expression from the shuttle vector, TSB was supplemented with xylose to a final concentration of 2% when cultures reached an OD_600_ of 0.3.

#### Nitrate Reduction Experiments.

For nitrate reduction measurements, strains were grown in TSB supplemented with sodium nitrate (Sigma-Aldrich, St. Louis, MO, USA) to a final concentration of 5 mM under microaerophilic conditions. Briefly, cultures were initially grown in 35mL of TSB, under aerobic conditions to an OD_600_ of 0.6. Then TSB supplemented with NaNO_3_ and xylose, to final concentrations of 5 mM and 2%, were added to a final volume of 75 mL. This represented 60% of the final capacity of the flasks. The flasks were sealed with parafilm to ensure oxygen limitation. *S. aureus*, grown for the creation of lawns for transduction or for single colonies, were grown on Tryptic Soy Agar (BD Biosciences, Franklin Lakes, NJ, USA) plates supplemented with chloramphenicol to a final concentration of 10 μg / mL as needed to select for plasmids used in this study. All *Escherichia coli* strains used in this study, for the purpose of cloning, were grown in SOC liquid media (New England Biolabs, Ipswich, MA, USA) or LB Lennox broth (KD Medical, Columbia, MD, USA) and transformants were selected for on LB (Lennox) Agar (BD Biosciences, Franklin Lakes, NJ, USA) plates supplemented with ampicillin to a final concentration of 100 μg / mL.

### Strains, Plasmids, and Oligonucleotides.

All strains and plasmids are listed in Table 1. *Staphylococcus aureus* strains SH1000 their derivatives used in this study and are listed in Table 1 (12). All plasmids used in this study are previously described derivatives of pEPSA5, including pEP-*sroA*, pEP-*sroB*, and pEP-*sroAB* and are all listed in Table 1 (13). Oligonucleotides used for PCR amplification, Real-Time Quantitative Reverse Transcription PCR (RT-qPCR) analysis, or as biotinylated Northern Blot probes are listed in Table 2.

### Total RNA Isolation for expression validation.

Total RNA was isolated using the FastRNA Pro^™^ Blue Kit (MP Biomedicals, Irvine, CA, USA). Briefly, *S. aureus* cells of interest were harvested from 5–50 mL of culture, following *sroA* induction for either 30 minutes or 3 hours. *S. aureus* cell pellets were resuspended in 1 mL of RNA Pro Solution, added to silica beads, in 2 mL tubes, and lysed using the Precellys^®^ 24 Dual Homogenizer (Bertin Corporation, Rockville, MD, USA) with the Cryolys for Precellys^®^ adaptor to ensure integrity of RNA following heat exposure. The homogenate was subjected to centrifugation and further processed using chloroform extraction and ethanol precipitation overnight at −80°C. RNA cell pellets were resuspended in 50–100 μL of RNase-free H_2_O. RNA concentration was measured using a Nanodrop OneC spectrophotometer (Thermo Fisher Scientific, Waltham, MA, USA).

### RNA Sequencing.

RNA sequencing and data analysis was performed as described previously (14). Briefly, wild type SH1000 carrying either empty pEPSA5 or pEP-*sroA*, were grown overnight as described in materials and methods. Next, these cultures were used to inoculate fresh TSB-Cm and allowed to grow for a further 3h. After this time, these cultures were used to seed fresh TSB at an OD_600_ of 0.05. Strains were allowed to grow until exponential phase (OD_600_ = 0.3) before the addition of xylose to a final concentration of 2%, to induce expression of *sroA*. These cultures were grown for 30 mins or 3 hours before samples were combined with 5 mL ice-cold PBS and subject to centrifugation. Total RNA extractions were performed using a Qiagen RNAeasy Kit and DNA was removed with the Ambion TURBO DNA-free kit. RNA quality was determined using an Agilent 2100 Bioanalyzer with an RNA 6000 Nano kit to confirm RNA integrity (RIN). Only samples with a RIN of 9.7 or higher were used in this study. Triplicate samples for each strain, from independently grown cultures, were then pooled at equal RNA concentrations, followed by rRNA removal using a MICROBExpress Bacterial mRNA enrichment kit. Efficiency of rRNA removal was confirmed using an Agilent 2100 Bioanalyzer with an RNA 6000 Nano kit. These mRNA samples were then subject library preparation using a Truseq Stranded mRNA Kit (Illumina) with the mRNA enrichment steps omitted. Fragment size, quantity and quality were assessed using an Agilent 2100 Bioanalyzer with an RNA 6000 Nano kit. Library concentrations for the pooling of barcoded samples was assessed with a KAPA Library Quantification kit. Samples were run on an Illumina NextSeq with a 150-cycle NextSeq Mid Output Kit v2.5. Experimental data from this study were deposited in the NCBI Gene Expression Omnibus (GEO) database (GEO accession number GSE329187). Data was exported from BaseSpace (Illumina) in fastq format and uploaded to CLC Genomics Workbench for analysis. Data was aligned to the NCTC 8325 reference genome file, reannotated to include newly discovered small RNAs (NC_007795.1) (15). Comparisons were carried out following quantile normalization via the Qiagen Bioinformatics experimental fold change feature.

### RT-qPCR transcriptional analysis:

To validate RNA-seq findings, a selection of genes were assayed by Real-Time Quantitative Reverse transcription PCR (RT-qPCR). Strains were grown under the indicated conditions, RNA was harvested, genomic DNA was removed, and RNA quality was assessed as described above. The High-Capacity cDNA Reverse Transcription Kit with RNase Inhibitor (Applied Biosystems^™^) were used to reverse transcribed 500 ng of total RNA, following the manufacturer’s instructions. RT-qPCR was performed using gene-specific primers (Table 2) and PowerTrack SYBR Green Master Mix (Applied Biosystems^™^ Foster City, CA, USA). Reactions were run on an Applied Biosystems StepOnePlus^™^ Real-Time PCR System, and SYBR Green fluorescence detection. The thermal cycling conditions consisted of an initial denaturation at 98 °C for 10 min, followed by cycling between 98 °C and 60 °C. Melt-curve analysis was performed at the end of each run to confirm amplification specificity. Negative controls (No template) were included on each plate. Levels of gene expression were normalized to the level of 16S rRNA gene as an internal control, and fold change of expression was assessed for each target gene overexpression relative to wild-type samples using the 2^−ΔΔCt^ method. Finally, data were analyzed using StepOne^™^ Software (Applied Biosystems^™^, Foster City, CA, USA).

### Nitrate Metabolism Analysis.

*Staphylococcus aureus* SH1000 containing pEPSA5 or pEP-*sroA* were grown aerobically to mid-exponential phase (OD_600_ of 0.3), after which cultures were shifted to microaerophilic or anaerobic growth conditions. Cultures were supplemented with xylose to a final concentration of 2% to induce *sroA* expression, and 5mM final concentration of NaNO3 at the final OD of 0.3. Flasks were tightly sealed using parafilm to create the microaerobic conditions. Samples were collected at 0, 2, 4, 6, 8, and 20 hours and subjected to centrifugation to separate the supernatant, which were stored at −20°C, until further utilized. The supernatant was further diluted with Nitrate/Nitrite assay buffer or DEPC water for nitrate/nitrite metabolite analysis. Total Nitrate and Nitrite concentrations were determined using a Griess reagent–based colorimetric assay (Nitrate/Nitrite Colorimetric Assay Kit, Cayman Chemical, Ann Arbor, MI, USA), and absorbance was measured at 540–550 nm using a SpectraMax iD5 Multi-Mode Microplate Reader (Molecular Devices, San Jose, CA, USA).

### Statistical Analysis.

All statistical analysis was executed using Prism 9 software (GraphPad, San Diego, CA, USA). Statistical significance for qRT-PCR was determined using one-way Analysis of Variance (ANOVA) followed by Tukey’s multiple-comparisons test (ns – not significant, *P < 0.05, **P < 0.01, ***P < 0.001, ****P < 0.0001).

## Results

### SroA overexpression rewires the *S. aureus* Transcriptome.

**Figure 1.**
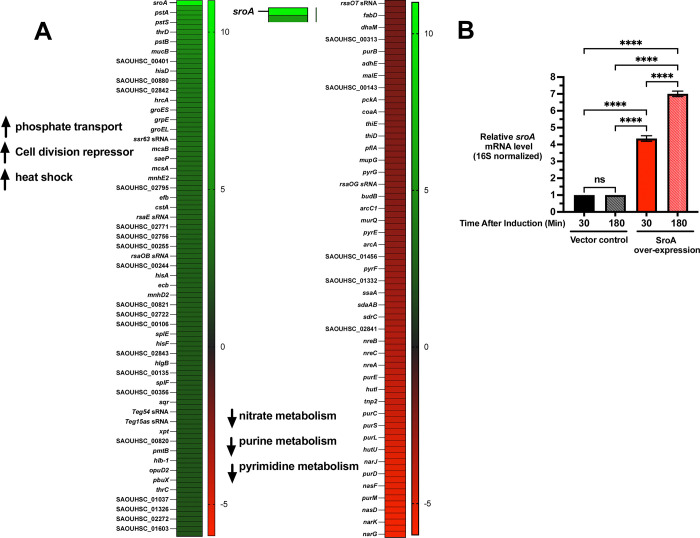
SroA Overexpression Induces Global Transcriptome Remodeling. (A) Heatmaps showing genes significantly upregulated (left) or downregulated (right) following xylose-induced overexpression of *sroA* in *Staphylococcus aureus* (false discovery rate [FDR] < 0.05, fold change > 2). (B) Quantitative RT-PCR analysis confirming induction of *sroA* transcripts 30 and 180 minutes after induction relative to vector control cultures. Transcript levels were normalized to 16S rRNA. Data represent mean ± SEM from three biological replicates. Statistical significance was determined using one-way ANOVA with Tukey’s multiple-comparisons test (ns = not significant, *P < 0.05, **P < 0.01, ***P < 0.001, ****P < 0.0001).

To define the global regulatory role of SroA and further investigate its relationship to SigS-dependent stress adaptation in *S. aureus*, we performed RNA sequencing (RNA-seq) to characterize transcriptome dynamics following overexpression of episomally encoded *sroA* in *Staphylococcus aureus* strain SH1000. Using a previously described xylose-inducible allele of *sroA*, rRNA-depleted total RNA from cultures overexpressing *sroA* was compared to rRNA-depleted total RNA from empty vector control cultures, after 180 minutes of xylose-induction initiated in early log-phase of growth. RNA-seq analysis revealed extensive remodeling of the *S. aureus* SH1000 transcriptome following SroA overexpression. Approximately 200 transcripts exhibited changes of at least twofold relative to the control strain (Fig. 1A; Table S1 – S3), indicating that SroA exerts broad regulatory effects across the *S. aureus* transcriptome. Both upregulated and downregulated genes were identified, suggesting that SroA influences multiple regulatory pathways rather than acting exclusively as an activator or repressor. Quantitative RT-PCR analysis confirmed robust induction of *sroA* transcripts following xylose treatment relative to the vector control (Fig. 1B), validating the experimental conditions used for transcriptomic profiling.

Inspection of the modulated transcripts revealed several functional gene categories affected by SroA overexpression. Genes involved in central metabolic processes, including nitrate respiration and nucleotide biosynthesis, were among the most strongly repressed. In contrast, transcripts associated with stress responses and nutrient acquisition represent notable gene clusters among the upregulated gene set. Notably, several genes encoding heat shock chaperones and protein-folding factors were modestly induced, a response that may reflect cellular adaptation to increased protein production resulting from inducible *sroA* expression rather than direct transcriptional regulation by SroA. Together, these results suggest that SroA overexpression promotes a broad reprogramming of metabolic and stress-response pathways during conditions in which SigS-dependent regulatory circuits are active. To further assess the reliability of the RNA-seq dataset, representative transcripts from highly modulated operons or functional gene classes were examined by quantitative RT-PCR. These experiments confirmed the directional changes observed in the RNA-seq dataset, supporting the validity of the transcriptomic analysis and providing independent verification of the major transcriptional trends identified in the sequencing data. While these studies utilized inducible overexpression, the coordinated nature of transcriptional changes across defined metabolic and stress-response pathways argues against nonspecific effects of protein overproduction

### SroA represses the nitrate reductase operon.

Consistent with broader transcriptomic trends, nitrate respiration genes were among the most strongly repressed transcripts following SroA overexpression were genes encoding the nitrate reductase system. RNA-seq analysis revealed pronounced repression of genes within the *narGHJI-nreABC locus*, which includes the *narGHJI* operon encoding the catalytic components of the nitrate reductase complex and the *nreABC* regulatory genes that control nitrate respiration. Transcripts within the *narGHJInreABC* locus were reduced between approximately 12- and 50-fold relative to vector control conditions (Table S1and S2). While expression of the redox-responsive regulator *rex* remained largely unchanged, the response regulator *nreC*, which contributes to regulation of nitrate respiration genes, was reduced approximately 13-fold (Table S1and S2).

**Figure 2.**
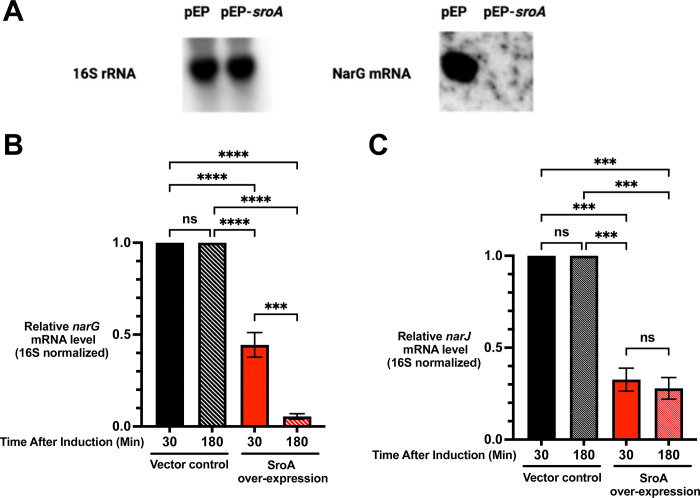
SroA represses expression of the nitrate reductase operon (*narG*-*narJ*). (A) Northern blot analysis of *narG* transcripts following induction of *sroA* from a xylose-inducible promoter compared with the empty vector control. Total RNA was harvested 180 minutes after induction. 16S rRNA is shown as a loading control. (B) Quantitative RT-PCR analysis of *narG* transcript levels 30 and 180 minutes after induction of *sroA* relative to vector control cultures. (C) Quantitative RT-PCR analysis of *narJ* transcript levels 30 and 180 minutes after induction of *sroA* relative to vector control cultures. Transcript levels were normalized to 16S rRNA. Data represent mean ± SEM from three biological replicates. Statistical significance was determined using one-way ANOVA with Tukey’s multiple-comparisons test (ns = not significant, *P < 0.05, **P < 0.01, ***P < 0.001, ****P < 0.0001)

To validate the RNA-seq results, expression of the nitrate reductase operon was examined using both Northern blotting and quantitative RT-PCR. Northern blot analysis revealed a marked reduction in *narG* transcript abundance following overexpression of *sroA* relative to the vector control, while 16S rRNA levels remained unchanged, confirming equal RNA loading (Fig. 2A). Quantitative RT-PCR analysis further confirmed repression of *narG* transcripts following induction of *sroA*. *narG* transcript levels, normalized to 16S rRNA, were reduced approximately 3-fold and 20-fold after 30 and 180 minutes of induction, respectively, relative to vector controls (Fig. 2B). These differences were statistically significant (*P* < 0.0001, one-way ANOVA with Tukey’s multiple-comparisons test).

Similarly, *narJ*, a downstream gene within the nitrate reductase operon, was also repressed. narJ transcript levels, normalized to 16S rRNA, were reduced approximately 3-fold after both 30 and 180 minutes of induction relative to vector controls (Fig. 2C). These differences were statistically significant (*P* < 0.001, one-way ANOVA with Tukey’s multiple-comparisons test). Notably, repression of *narG* became more pronounced at the later time point, whereas *narJ* repression remained similar at both time points.

This pattern suggests that SroA-dependent repression across the nar operon may be more pronounced at the 5′ end of the transcript. Together, these results confirm the RNA-seq observation that SroA strongly represses transcription of the nitrate reductase operon and suggest that SroA may influence nitrate respiration and associated redox metabolic processes. Because the *narGHJI* operon encodes the catalytic machinery required for nitrate respiration, we next examined whether repression of these transcripts corresponded to measurable changes in nitrate metabolism.

### SroA represses nitrate respiration activity and alters nitrate–nitrite dynamics.

**Figure 3.**
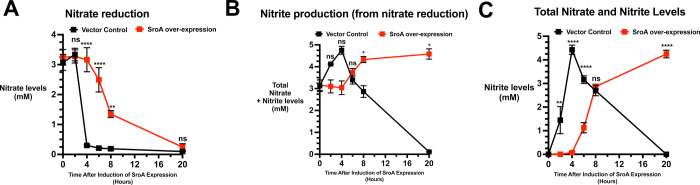
SroA alters nitrate and nitrite dynamics during nitrate metabolism. Nitrate and nitrite levels were quantified over time using a colorimetric assay following induction of *sroA* overexpression under oxygen-limited conditions. (A) Nitrate levels over time in vector control and *sroA*-overexpressing cultures. (B) Nitrite levels over time in vector control and *sroA*-overexpressing cultures. (C) Total nitrate plus nitrite levels over time. Data represent mean ± SEM from at least three independent biological replicates. Statistical significance was determined using two-way ANOVA with Šidák’s multiple comparisons test (ns = not significant, *P < 0.05, **P < 0.01, ***P < 0.001, ****P < 0.0001)

To determine whether repression of the *nar* operon corresponded to measurable changes in nitrate metabolism, nitrate reduction was quantified using a colorimetric nitrate/nitrite assay following induction of *sroA* expression. Cultures overexpressing *sroA* exhibited delayed depletion of nitrate compared to vector control cultures (Fig. 3A), with approximately 10-fold and 6-fold higher nitrate levels remaining at 4 and 8 hours, respectively, indicating impaired nitrate reduction over time. Consistent with this, nitrite dynamics were also altered in *sroA-*overexpressing cultures, including delayed production and altered accumulation patterns at later time points (Fig. 3B). At early time points, nitrite levels were markedly reduced, with an approximately 6-fold decrease at 4 hours and minimal detectable nitrite in *sroA*-overexpressing cultures compared to ~4.5 mM in vector control. Nitrite levels remained significantly reduced at 6 hours (~3-fold decrease), while differences were not significant at 8 hours, and by 20 hours nitrite was undetectable in *sroA*-overexpressing cultures but remained elevated (~4.5 mM) in control cultures. Measurement of total nitrate plus nitrite confirmed that these changes reflect altered metabolic flux rather than differences in total nitrogen recovery (Fig. 3C). Notably, at 20 hours total nitrate plus nitrite remained elevated in *sroA*-overexpressing cultures but was largely depleted in vector control, consistent with accumulation of nitrite and incomplete reduction of nitrogen intermediates. These results demonstrate that SroA overexpression impairs nitrate respiration and disrupts the temporal dynamics of nitrate-to-nitrite conversion. Together, these findings indicate that SroA modulates respiratory metabolism under oxygen-limited conditions, although the underlying regulatory mechanism remains to be defined.

### SroA Represses de novo purine and pyrimidine biosynthesis.

SroA overexpression led to potent repression of genes involved in both purine and pyrimidine biosynthesis. RNA-seq analysis revealed strong downregulation of the PurR-regulated *purEKCSQLFMNHD* operon, which encodes multiple enzymes required for de novo purine nucleotide biosynthesis.

Transcripts within this operon were reduced approximately 17–40-fold relative to vector control conditions, while transcript levels of the transcriptional regulator *purR* itself remained unchanged (Table S1 and S2). In addition to this operon-level repression, several non-operonic purine biosynthetic genes were also suppressed, including *purB* (~4-fold), *guaC* (~18-fold), and *purA* (~35-fold) (Table S1and S2). Genes involved in pyrimidine biosynthesis were similarly affected. Transcripts within the *pyrPBCcarABpyrFE* locus, which encode enzymes responsible for pyrimidine nucleotide synthesis and transport, were reduced between 5- and 20-fold relative to vector controls (Table S1and S2).

**Figure 4.**
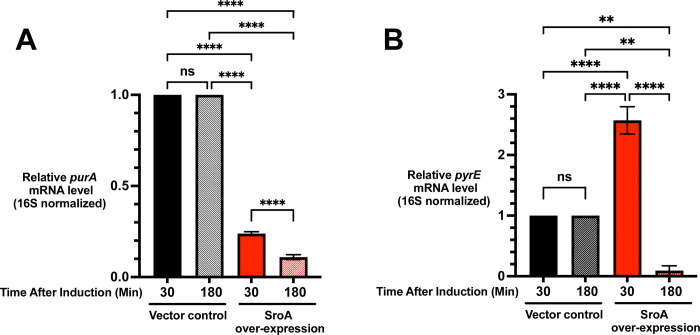
SroA represses purine and pyrimidine biosynthesis genes. Quantitative RT-PCR analysis of representative genes from the *pur* (*purA*) and *pyr* (*pyrE*) operons following overexpression of *sroA*. These operons encode enzymes involved in de novo purine and pyrimidine biosynthesis. Transcript levels were measured 30 and 180 minutes after induction of *sroA* and compared with vector control cultures. Expression values were normalized to 16S rRNA. Data represent mean ± SEM from three biological replicates. Statistical significance was determined using one-way ANOVA with Tukey’s multiple-comparisons test (ns = not significant, *P < 0.05, **P < 0.01, ***P < 0.001, ****P < 0.0001) (A) *purA* transcript levels. (B) *pyrE* transcript levels.

To validate the RNA-seq observations, expression of representative genes from the purine and pyrimidine biosynthetic pathways was examined by quantitative RT-PCR. Consistent with the transcriptomic analysis, expression of *purA* was significantly reduced following induction of *sroA*, with transcript levels decreasing approximately 5-fold after 30 min and 10-fold after 180 min relative to vector controls (Fig. 4A). In contrast, *pyrE* exhibited a time-dependent response. Following 30 min of *sroA* induction, *pyrE* transcript levels increased approximately 2.5-fold relative to vector controls (*P* < 0.0001), whereas after 180 min of induction, *pyrE* transcript levels were reduced approximately 20-fold (*P* < 0.01) (Fig. 4B). These results indicate that repression of nucleotide biosynthetic genes following SroA overexpression may be temporally dynamic, potentially reflecting secondary regulatory responses triggered by prolonged SroA activity.

Together, these findings indicate that SroA overexpression broadly perturbs nucleotide biosynthetic pathways. Because nucleotide synthesis is tightly linked to cellular growth and replication, repression of these pathways may reflect a metabolic shift that limits biosynthetic activity under conditions in which SroA is highly expressed.

### SroA activates phosphate transport and SOS response genes.

**Figure 5.**
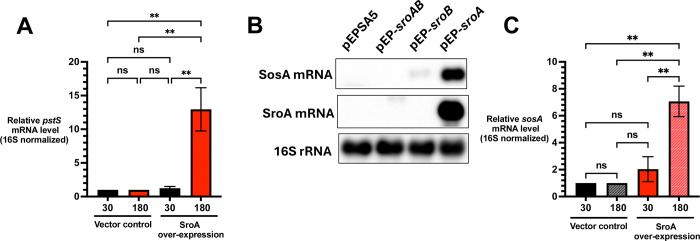
SroA activates stress response and phosphate transport genes. Quantitative RT-PCR and Northern blot analyses were performed to examine transcriptional changes following overexpression of *sroA*. Transcript levels were normalized to 16S rRNA. Data represent mean ± SEM from three biological replicates. Statistical significance was determined using one-way ANOVA with Tukey’s multiple-comparisons test (ns = not significant, *P < 0.05, **P < 0.01, ***P < 0.001, ****P < 0.0001). (A) Quantitative RT-PCR analysis of *pstS* transcript levels 30 and 180 minutes after induction of *sroA* relative to vector control cultures. (B) Northern blot analysis of *sosA* transcripts in strains harboring pEPSA5 (vector), pEPSA5-*sroA*, pEPSA5-*sroB*, or pEPSA5-*sroAB*. Total RNA was harvested 180 minutes after induction. 16S rRNA is shown as a loading control. (C) Quantitative RT-PCR analysis of *sosA* transcript levels 30 and 180 minutes after induction of *sroA* relative to vector control cultures.

Transcriptome analysis revealed that several stress response and nutrient acquisition genes were upregulated following SroA overexpression. Among the most strongly induced transcripts were components of the phosphate-specific transport system as well as genes associated with the SOS response (Table S1and S3). RNA-seq analysis revealed increased expression of genes within the *pstSCAB operon*, including *pstS*, which encodes the phosphate-binding component of the high-affinity phosphate transport system. Quantitative RT-PCR analysis confirmed that *pstS* transcript levels increased approximately 12-fold on average following induction of sroA relative to vector control cultures (*P* < 0.01) (Fig. 5A). These findings indicate that SroA overexpression promotes transcription of genes involved in phosphate acquisition. Upregulation of phosphate transport systems is consistent with adaptation to nutrient limitation and stress-associated metabolic remodeling.

In addition to phosphate transport genes, transcripts associated with the SOS response were also increased following SroA overexpression. The *sosA* gene, which encodes a cell division inhibitor associated with DNA damage responses, exhibited elevated transcript levels in the RNA-seq dataset (Table S1 and S3). Northern blot analysis confirmed increased *sosA* transcript abundance following overexpression of *sroA* relative to the vector control (Fig. 5B). Quantitative RT-PCR further demonstrated that mean *sosA* transcript levels increased approximately 2-fold and 7-fold after 30 and 180 minutes of *sroA* induction, respectively (*P* < 0.01) (Fig. 5C). Together, these results confirm that SroA overexpression promotes transcription of SOS-associated genes.

## Discussion

Together, these findings identify a previously unrecognized role for SroA in regulating respiratory metabolism and stress-responsive pathways in *Staphylococcus aureus*. Transcriptomic analysis revealed that inducible expression of *sroA* results in substantial remodeling of the *S. aureus* transcriptome, affecting genes involved in central metabolism, stress responses, and nutrient acquisition. Among the most strongly affected pathways were those associated with nitrate respiration and nucleotide biosynthesis, while transcripts involved in phosphate acquisition and the SOS response were induced. Together, these findings suggest that SroA participates in coordinating metabolic and stress-response pathways that allow *S. aureus* to adapt to changing environmental conditions.

One of the most striking observations from the transcriptomic analysis was the strong repression of the nitrate reductase locus following SroA overexpression. Genes within the *narGHJI-nreABC* region, which encode the catalytic machinery and regulatory components required for nitrate respiration in Staphylococcal species (16–19), were among the most highly repressed transcripts in the dataset. Independent validation by Northern blotting and quantitative RT-PCR confirmed that expression of *narG* and *narJ* was significantly reduced following induction of *sroA*. Importantly, repression of these transcripts corresponded with decreased nitrate reduction activity in cultures overexpressing SroA, indicating that transcriptional repression of the nitrate reductase operon has measurable physiological consequences for nitrate respiration. These findings identify SroA as a previously unrecognized regulator of respiratory metabolism in *S. aureus*. Whether SroA influences the nitrate reductase operon directly or through secondary regulatory pathways remains to be determined.

Respiratory flexibility is an important feature of bacterial adaptation during infection. Within host tissues, oxygen availability can vary widely depending on the infection site, inflammatory responses, and host metabolic activity. Under conditions of oxygen limitation, *S. aureus* can utilize alternative electron acceptors such as nitrate to maintain redox balance and energy production. Regulation of nitrate respiration therefore represents an important mechanism by which the bacterium adjusts its metabolism in response to environmental constraints. The observation that SroA represses expression of the nitrate reductase operon suggests that SroA functions as a negative regulator of respiratory adaptation under oxygen-limited conditions.

In addition to its effects on nitrate respiration, SroA overexpression resulted in repression of genes involved in de novo nucleotide biosynthesis. Multiple genes within the PurR-regulated purine biosynthetic operon and several additional purine metabolism genes were strongly downregulated in the transcriptomic dataset, and quantitative RT-PCR confirmed repression of representative genes within these pathways. Genes associated with pyrimidine biosynthesis were similarly affected, although some transcripts exhibited temporal differences in their response to SroA overexpression. Because nucleotide biosynthesis is tightly coupled to cellular growth and DNA replication, repression of these pathways may reflect a broader shift toward reduced biosynthetic activity under conditions in which SroA is highly expressed.

Conversely, several stress-response and nutrient acquisition pathways were induced following SroA overexpression. Transcripts within the *pstSCAB* phosphate transport operon were strongly increased, and quantitative RT-PCR confirmed elevated expression of *pstS*, suggesting enhanced phosphate acquisition under these conditions (20–22). Phosphate uptake systems are known to contribute to *Staphylococcus aureus* fitness in phosphate-limited and host-associated environments, including during nutritional immunity and resistance to host-derived stresses such as nitric oxide (20–23). In addition, transcripts of the SOS-associated gene *sosA*, which encodes a cell division inhibitor involved in DNA damage responses (24, 25), were also elevated. Induction of SOS-associated genes may reflect activation of cellular pathways that promote survival under conditions that threaten genomic integrity. The simultaneous modulation of metabolic pathways and stress-response genes suggests that SroA may coordinate broader physiological adaptations in *Staphylococcus aureus* that extend beyond the canonical SigS-mediated stress response circuit. Together, these responses are consistent with a shift toward a stress-adaptive, growth-limited physiological state. Collectively, these findings support a model in which SroA promotes a coordinated physiological shift characterized by repression of energy-intensive metabolic pathways, including nitrate respiration and nucleotide biosynthesis, and activation of stress adaptation and nutrient acquisition systems.

**Figure 6.**
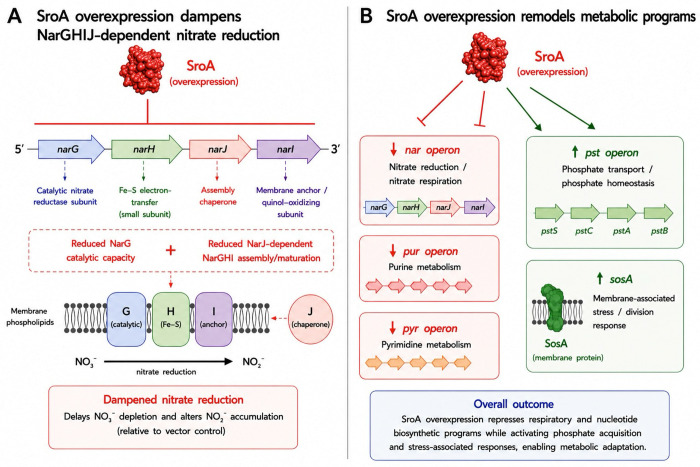
Proposed model for SroA-mediated repression of nitrate reduction and broader metabolic remodeling. (A) Model depicting how SroA overexpression dampens NarGHIJ-dependent nitrate reduction under oxygen-limited, nitrate-replete conditions. The *narG-narH-narJ-narI* operon encodes the multi-unit catalytic nitrate reductase subunit. Repression of the *nar* operon is predicted to reduce NarG catalytic capacity, resulting in dampened nitrate reduction, delayed nitrate depletion, and altered nitrite accumulation relative to vector control. (B) Broader model of SroA-dependent transcriptional remodeling. qPCR analysis showed that SroA overexpression represses the *nar*, *pur*, and *pyr* operons while activating the *pst* operon and *sosA*. These changes suggest that SroA coordinates repression of nitrate respiration and nucleotide biosynthetic programs with activation of phosphate acquisition and membrane-associated stress/division responses. Red blunt-ended lines indicate repression or decreased transcript abundance, and green arrows indicate activation or increased transcript abundance.

Finally, the regulatory role of SroA may be considered in the context of its relationship to the stress-responsive sigma factor SigS. Previous work demonstrated that the *sroAB* operon is a direct transcriptional target of SigS and that the small proteins SroA and SroB exert opposing regulatory effects on *sigS* expression (11). In particular, SroA was shown to promote accumulation of sigS mRNA, suggesting that SroA participates in a feedback mechanism that stabilizes sigS transcripts and amplifies SigS-dependent stress signaling (11). The results presented here extend this regulatory relationship by demonstrating that SroA also influences broader metabolic pathways in *Staphylococcus aureus*, including repression of nitrate respiration and modulation of nucleotide biosynthetic genes. Given that SroA is directly induced by SigS, these findings suggest that SigS-dependent signaling extends beyond canonical stress responses to include coordinated metabolic restraint. Because SigS is activated in response to genotoxic and cell envelope stress, and *sroA* expression is specifically induced in a SigS-dependent manner under genotoxic conditions (11), the regulatory effects of SroA described here likely occur within the broader SigS stress-response network. In this context, SigS-dependent signaling may contribute to a coordinated checkpoint that integrates metabolic restraint with DNA damage–associated cell division control, consistent with induction of SOS-associated factors such as *sosA* and repression of nucleotide synthesis to potentially facilitate DNA repair. Such a mechanism could allow *S. aureus* to transiently modulate respiratory metabolism while prioritizing stress adaptation and cellular repair. These observations support a model in which SigS-dependent induction of SroA links stress-responsive signaling to repression of nitrate respiration and broader metabolic remodeling in *S. aureus* (Fig. 6). Together, these findings expand the regulatory landscape of SigS-dependent stress signaling and reveal a previously unrecognized connection between stress-responsive pathways and respiratory metabolic control in *S. aureus*.

## Figures and Tables

**Table 1 -:** Strains and Plasmids

Strains		

Strain Number	Bacteria / Genotype	Source or reference
SH1000	wild type laboratory strain: 8325–4 *rsbU* ^+^	Lab collection
	SH1000 pEPSA5	(11)
	SH1000 pEP-*sroA*	(11)
	SH1000 pEP-*sroB*	(11)
	SH1000 pEP-*sroAB*	(11)

Plasmids		

Plasmid Number	Characteristics	Source or reference
pEPSA5	*E. coli* / S. aureus shuttle plasmid vector p15A *ori* in *E. coli*; *bla* (Amp^R^) in *E. coli*; *cat* (Cm^R^) in *S. aureus*. 6852 bp	Lab collection
pEP-*sroA*	*sroA* gene cloned into the EcoRI / PstI site of pEPSA5; *bla* (Amp^R^) in *E. coli*; *cat* (Cm^R^) in *S. aureus*	(11)
pEP-*sroB*	*sroB* gene cloned into the EcoRI / PstI site of pEPSA5; *bla* (Amp^R^) in *E. coli*; cat (Cm^R^) in *S. aureus*	(11)
pEP-*sroAB*	*sroAB* gene cloned into the EcoRI / PstI site of pEPSA5; *bla* (Amp^R^) in E. coli; cat (Cm^R^) in *S. aureus*	(11)

**Table 2 -:** Synthetic Oligonucleotides used as primers or probes

Oligo #	Sequence	Purpose
*narG* FWD Set 2	GGAGCCACGATAAAGAAGTAAGA	qRT-PCR of *narG* in *S. aureus* F
*narG* REV Set 2	CCGGACCACAACTTGGATAG	qRT-PCR of *narG* in *S. aureus* R
*narJ* FWD Set 1	AAAGAACGTGGGCAAATGTTAG	qRT-PCR of *narJ* in *S. aureus* F
*narJ* REV Set 1	GCATCAACGGTAGATAATCAGAAAG	qRT-PCR of *narJ* in *S. aureus* R
*pstS* FWD Set 1	AACAAAGAGGGAATCGGATACTT	qRT-PCR of *pstS* in *S. aureus* F
*pstS* REV Set 1	TCGTAGGCTCTGTTGCTTTAC	qRT-PCR of pstS in *S. aureus* R
*purA* qPCR-for	CCCGTGATGGGTAAGGTGA	qRT-PCR of *purA* in *S. aureus* F
*purA* qPCR-rev	GGCACATCAATTATCAGTTGCGA	qRT-PCR of *purA* in *S. aureus* R
*pyrE* qPCR-for	CAGCTGGTATTCCACATGCAGC	qRT-PCR of *pyrE* in *S. aureus* F
*pyrE* qPCR-rev	CTGCTGTGACTGAAGATCCCC	qRT-PCR of *pyrE* in *S. aureus* R
SA-16S qPCR-for-2	TCACCGTAGCATGCTGATCT	qRT-PCR of 16S in *S. aureus* F
SA-16S qPCR-rev-2	AGGTGGGGATGACGTCAAAT	qRT-PCR of 16S in *S. aureus* R
*sosA* FWD Set 3	TGTACGAAATGACTGACCATCAA	qRT-PCR of *sosA* in *S. aureus* F
*sosA* REV Set 3	AGCGAACACTTTCCCATCTC	qRT-PCR of *sosA* in *S. aureus* R
*sroA* FWD Set 2	AGTAGACGCTAACGGCAAAC	qRT-PCR of *sroA* in *S. aureus* F
*sroA* REV Set 2	CGCTCAATAAGTTTGCTGAATGT	qRT-PCR of *sroA* in *S. aureus* R
Probe name	Sequence	Purpose
*narG* probe	GCCTGTACAGTTAACACCATGTGTTGTTCTTACTTC	biotinylated probe for northern blot analysis of *narG*
*sosA* probe	CGTTGTGACAATGTATCAATTTATTAAAGCGAACAC	biotinylated probe for northern blot analysis of *sosA*
